# Rheology of Crumb Rubber-Modified Warm Mix Asphalt (WMA)

**DOI:** 10.3390/polym16070906

**Published:** 2024-03-26

**Authors:** Ghazi G. Al-Khateeb, Alaa Sukkari, Helal Ezzat, Eyad Nasr, Waleed Zeiada

**Affiliations:** 1 Department of Civil and Environmental Engineering, College of Engineering, University of Sharjah, Sharjah 27272, United Arab Emirates; u18200507@sharjah.ac.ae (A.S.); u20200169@sharjah.ac.ae (E.N.); wzeiada@sharjah.ac.ae (W.Z.); 2 Department of Civil Engineering, Jordan University of Science and Technology, Irbid 22110, Jordan; 3 Research Institute of Sciences and Engineering, University of Sharjah, Sharjah 27272, United Arab Emirates; hhelal@sharjah.ac.ae; 4 Civil Engineering Department, Faculty of Engineering, Delta Higher Institute for Engineering and Technology, Mansoura 35681, Egypt; 5 Public Works Engineering Department, Faculty of Engineering, Mansoura University, Mansoura 35516, Egypt

**Keywords:** WMA, rheology, crumb rubber, sasobit, zycotherm, Dynamic Shear Rheometer, asphalt binder, performance

## Abstract

This study explores the impact of adding waste vehicular crumb rubber to the commercially available warm mix additives Sasobit^®^ and Zycotherm^®^ on modified asphalt binders’ physical and rheological properties. Various concentrations of crumb rubber (0%, 10%, 15%, and 20%) were introduced to asphalt binder samples with 2% and 4% Sasobit and 1.5% and 3% Zycotherm. The investigation employed conventional tests (penetration and softening point) and advanced mechanical characterization tests, including Superpave rotational viscosity (RV), Dynamic Shear Rheometer (DSR), DSR multi-stress creep recovery (MSCR), DSR linear amplitude sweep (LAS), and Bending Beam Rheometer (BBR). Traditional tests measured the asphalt consistency, while workability was assessed through the RV test. The results showed that the Zycotherm binders experienced a more significant penetration reduction than the Sasobit binders. Additionally, an increased crumb rubber content consistently elevated the softening point and rotational viscosity, enhancing the complex shear modulus (G*) values. Rubberized binders exhibited an improved rutting performance and low-temperature PG grades. Increasing the crumb rubber content enhanced fatigue life, with Z1.5CR20 and S2CR20 demonstrating the longest fatigue lives among the Zycotherm and Sasobit binders, respectively. Overall, Z1.5CR20 is recommended for colder climates, while S2CR20 is suitable for hot-climate applications based on extensive analysis.

## 1. Introduction

Hot mix asphalt (HMA) requires high temperature ranges for production, incurring high energy consumption with increased costs and carbon emissions [1]. Increases in energy and materials costs and the impact of environmental conditions on asphalt pavements in the last two decades have prompted the industry to assess the use of sustainable materials to enhance the performance of asphalt binders through the use of polymers [1,2] and bio-ash [3]. Other technologies that are currently being investigated are the use of recycled asphalt binders (RAB) [4], recycled asphalt pavements (RAP) [5], and warm mix asphalt (WMA) mixes. WMA technologies are used to lower the mixing temperature by 20 °C to 40 °C and improve the workability of asphalt binders to decrease the costs required to mix the aggregates with the binder using WMA additives [6]. Chemical WMA additives differ in their modification mechanism; therefore, careful selection of the WMA additive is required. Overall, WMA technology usage has been proven to decrease environmental impact while enhancing the performance of asphalt pavements [7]. 

Organic WMA additives consist of wax, manufactured using the Fischer–Tropsch (FT) method, which is added to reduce the viscosity of the asphalt binder during mixing, thus reducing the temperature needed for the production process [7]. One of the most well-known wax additives is Sasobit. Sasol Germany manufactures Sasobit, a natural organic wax produced from the Fischer–Tropsch method, which involves a process of natural gas liquefication [8]. Sasobit’s modifying mechanism relies on its melting point of 100 °C. When Sasobit is added to an asphalt binder heated above the wax’s melting temperature, it liquefies; thus, the viscosity of the asphalt binder is reduced. Cooling the asphalt binder crystalizes the wax, which increases the stiffness of the asphalt binder at performance temperature ranges [9,10]. The addition of Sasobit has been proven to increase the penetration and softening point while decreasing the viscosity of the asphalt binder properties [11,12,13]. Since Sasobit crystallizes in the asphalt binder when cooled, its rheological properties are enhanced. Studies have shown that, when increasing the Sasobit content, the high-temperature performance grade (PG) increases [14,15], and the non-recoverable creep compliance (J_nr_) decreases, i.e., increasing the traffic level at any given temperature grade [16,17,18]. As for intermediate temperatures and fatigue resistance, Sasobit has shown the potential to enhance fatigue resistance when a lateral amplitude sweep test (LAS) is carried out [19,20]. However, multiple studies have shown a slightly high susceptibility to low-temperature cracking, using a bending beam rheometer (BBR), of Sasobit-modified asphalt binders due to the increased stiffness of the binders [21,22]. 

Chemical WMA additives enhance the workability of asphalt binders at the microscopic level. These additives improve the adhesion between the aggregates and the asphalt binder, thus strengthening the interface [23,24]. Unlike organic WMA additives, the chemical WMA impact on the physical and rheological properties of the asphalt binder is not significant [25,26,27]. Studies have shown that WMA additives might reduce the asphalt binder’s high-temperature PG and rutting resistance [28,29]. One of the WMA additives that is currently being investigated is Zycotherm. Zycotherm, manufactured by Zydex^®^, India, is a nano-based antistrip additive that improves the workability of an asphalt binder by acting as a water repellent, thus improving the adhesion between the aggregates and the asphalt binder. While Zycotherm addition to an asphalt binder does not significantly improve the asphalt’s properties, Zycotherm has been shown to be potent in resisting moisture damage compared to other WMA additives [30,31,32]. 

Since WMA additives reduce viscosity and improve the workability of asphalt binders, WMA additives can be added to modifiers that increase the viscosity of asphalt binders, notably, crumb rubber modifiers (CRM). When crumb rubber is added to an asphalt binder, the rubber swells to 3–5 times its size, thus absorbing the maltenes in the asphalt binder, increasing the asphaltenes ratio [33]. This increase in the asphaltenes ratio results in a better performance of asphalt binders, given that the mixing conditions, material quantity and quality, and mixing process (dry or wet) are optimized [33]. Adding CRM to asphalt binders has been proven to enhance their physical and rheological properties and increase their resistance to rutting deformation, fatigue, and thermal cracking. While the CRMB’s performance has been observed [34,35,36], the compatibility between the asphalt binder and CRM and workability is challenging due to phase separation. [37,38]. To overcome the low workability of CRMB, CRM can be added to warm asphalt binders. The resulting mix performance can be further enhanced when incorporating CRM with WMA additives [39]. WMA and CRM added to asphalt mixtures exhibited a decrease in moisture damage susceptibility [40,41].

Adding CRM to warm asphalt binders prepared using Sasobit has shown an improved high-temperature performance, low-temperature thermal cracking resistance, and fatigue cracking resistance while decreasing viscosity [42,43,44]. In addition, when adding WMA and CRM to asphalt mixtures, the rutting resistance improved [45] and moisture damage susceptibility decreased [46]. CRM added to a warm binder prepared with chemical WMA additives weakens its rheological performance [47,48]. Combining the additives with the asphalt mixes showed a comparable performance to CRM-modified HMA at lower mixing and compaction temperatures [49]. 

## 2. Significance of Study

The current literature lacks a thorough investigation into the performances of chemical additives beyond Evotherm^®^. As a result, there is a clear need to conduct a comprehensive assessment of these additives when combined with crumb rubber. The goal is to understand how these combinations perform at various temperatures. This study aimed to thoroughly evaluate and draw comparisons between incorporating crumb rubber into asphalt binders modified with Sasobit and Zycotherm.

Furthermore, this study performed a comprehensive and detailed evaluation program on these modified asphalt binders. This involved the integration of warm mix additives and crumb rubber. Additionally, the experimental framework involved a wide range of both traditional and advanced rheological tests. The evaluation covered various aspects, including resistance to low-temperature cracking, intermediate-temperature fatigue performance, and the ability to withstand high-temperature rutting.

Additionally, it is important to note that the assessment plan in this study did not stop at these performance measurements, but also included considerations of consistency, workability, mixing and compaction temperatures, performance grading and classification, and advanced characterization tests for the modified asphalt binders at a wide range of temperatures, modifier ratios, strain levels, and loading frequencies.

## 3. Materials and Mixing Process

### 3.1. Asphalt Binder

This study utilized a 60/70 penetration-grade asphalt binder sourced from Shell Corporation, Hamburg, Germany. The asphalt binder’s properties, listed in Table 1, were examined. The Superpave Asphalt Binder Grading System was employed to determine its classification, resulting in a Superpave Performance Grade (PG) of PG64-22 for the asphalt binder.

### 3.2. Additives and Modification

**Figure 1:**
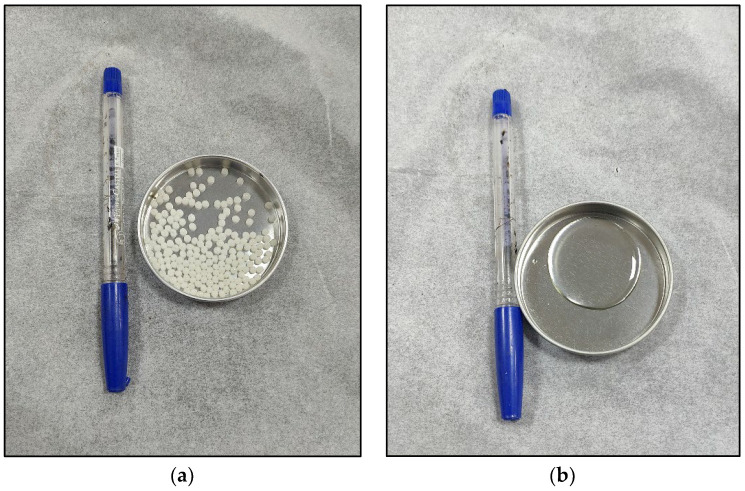
Warm mix additives (**a**) Sasobit and (**b**) Zycotherm.

This study utilized two types of warm mix additives: Sasobit, which is a wax-based additive manufactured through the Fischer–Tropsch method from natural gas and obtained from Sasol in, Johannesburg, South Africa, and Zycotherm, a chemical-based additive with nano-silane properties produced by Zydex Industries, Gujarat, India (Figure 1). To modify the asphalt binder, Sasobit was added at 2% and 4% by weight, while Zycotherm was added at 1.5% and 3%, based on the manufacturer’s recommendations. The properties of these additives are presented in Table 2, General Properties of the Warm Mix Additives. 

The crumb rubber used in this study was sourced from Beeah Recycling Center/Beeah Waste Management Company in Sharjah, United Arab Emirates. It was obtained by shredding waste (discarded) vehicle tires. The sieve analysis results of the crumb rubber are presented in Table 3, Sieve Analysis of Crumb Rubber.

### 3.3. Mixing Process

The mixing process involved combining each warm mix additive (Sasobit and Zycotherm) with the original asphalt binder using a high-shear mixer (ROSS Model: +100 LSI). This mechanical mixing was performed at a high shear speed of 1000 rpm and a mixing temperature of 150 °C for 30 min. The Sasobit was mixed with the asphalt binder at two different contents: 2% and 4% by weight of the asphalt binder. Similarly, the Zycotherm was mixed at contents of 1.5% and 3% by weight of the asphalt binder.

Furthermore, the warm mix asphalt binders were blended with crumb rubber using dispersion mixing geometry at a high shear speed of 2000 rpm and a mixing temperature of 170 °C for 60 min. Mixing the warm mix asphalt binder with the crumb rubber was carried out at three different crumb rubber contents: 10%, 15%, and 20% by weight of the asphalt binder. For convenience, throughout the paper, the asphalt mixes will be referred to using the abbreviations specified in Table 4.

### 3.4. Experimental Plan and Test Methods

The penetration test, ASTM D5 [50], examined the consistency of the asphalt binder by loading 100 g on a standard needle that penetrated the asphalt binder surface for 5 s while the sample was submerged in water at a temperature of 25 °C. The softening point, ASTM D36 [51], is the temperature at which a 3.5 g steel ball placed on top of a steel ring filled with the asphalt fell a 25 mm (1-in) distance at starting temperature of 4 ± 1 °C and a rate of heating 1 °C per minute. 

#### 3.4.1. Rotational Viscosity Test

ASTM D4402 [52] was conducted for the asphalt binders at a standard test temperature of 135 °C, representing the average mixing and laydown temperature for Hot Mix Asphalt (HMA) according to the Superpave specifications. In the RV test, a cylindrical spindle with a specified diameter and effective length rotated inside a container filled with the asphalt material to an appropriate height at a standard speed of 20 rpm. The dynamic (rotational) viscosity was measured using the RV device. This viscosity was calculated by dividing the shear stress by the shear strain rate. The shear stress was determined by measuring the torque needed to maintain a constant rotational speed, while the shear strain rate was obtained from the rotational speed using established equations. The RV, penetration, and softening point results will be used to calculate the mixing and compaction temperatures of the asphalt binders, as shown in Equation (1).

(1) $$\log\eta =10.5012\;-\;2.2601\log\left(\mathrm{PEN}\right)+0.00389{(\log\left(\mathrm{PEN})\right)}^{2}$$

#### 3.4.2. Performance Grade (PG) Test

The Performance Grade (PG) test, ASTM D6373 [53], measures the complex shear modulus (G*) value and the phase angle (δ) to obtain the rutting parameter (|G*|/Sin*δ*) at multiple temperatures. The test was performed over a temperature range from 64 °C to 82 °C, at increments of 6 °C. In this test, a sample with a diameter of 25 mm was placed in a Discovery Hybrid Rheometer (DHR), TA Instruments, New Castle, DE, USA, conditioned for 10 min at testing temperature, and subjected to a standard angular frequency of 10 rad/s (1.59 Hz) with the strain maintained at 12%. According to the Superpave specifications, the value of ((|G*|/Sin*δ*) should have a minimum value of 1.0 kPa for unaged asphalt binders and 2.2 kPa for short-term aged asphalt binders in rolling thin-film oven (RTFO), manufactured by Games Cox, San Diego, CA, USA, to pass a given temperature.

#### 3.4.3. Multiple-Stress Creep Recovery (MSCR) Test

The Multiple-Stress Creep Recovery (MSCR) test was performed according to the ASTM D7405 [54]. The MSCR test utilizes the well-established creep and recovery test concept to assess the potential for permanent deformation (rutting) in asphalt binders. It offers a more accurate high-temperature specification for asphalt binders that indicates their rutting performance accurately and is blind to modification. Using the DHR, a one-second creep load was applied to the asphalt binder sample. After the 1 s load was removed, the sample was allowed to recover (relax) for 9 s at a zero load. The MSCR test started with the application of low stress (0.1 kPa) for 10 creep/recovery cycles. Then, the stress was increased to 3.2 kPa and repeated for an additional 10 cycles. Therefore, the MSCR test measured the asphalt binder’s recovery and non-recoverable strain compliance. The asphalt sample used in the MSCR test was short-term aged in the RTFO test and had a diameter of 25 mm. The test was conducted at the original asphalt binder’s high-performance grade (PG) temperature (64 °C). The test data obtained were used to determine the recovered strain (γ_r_), the non-recoverable compliance (J_nr_), and the percent recovery (%R) at both stress levels for all tested asphalt binders.

#### 3.4.4. Linear Amplitude Sweep (LAS) Test

The Linear Amplitude Sweep (LAS) test, AASHTO T391 [55], is used to calculate the number of load cycles to fatigue failure. The test investigates fatigue resistance by utilizing cyclic loading to speed the damage. The rate of damage increase was used to determine the fatigue performance using predictive modelling techniques. The damage accumulation was calculated as per Equation (2):

(2) $$D(t)≅\sum_{i=1}^{N}{\left[\pi I_{D}\gamma_{0}^{2}\left(\left|G^{*}\right|Sin\delta_{i-1}-\left|G^{*}\right|Sin\delta_{i}\right)\right]}^{\frac{\alpha }{1+\alpha }}{\left(t_{i}-t_{i-1}\right)}^{\frac{1}{1+\alpha }}$$

where:

*I_D_* = initial value of │G*│ from the 1.0 percent applied strain interval; MPa

$\gamma_{0}$ = applied strain for a given data point, percent. 

|G*| = Complex Shear Modulus; MPa. 

*α* = reciprocal of m value

*t* = test time, seconds.

Based on the current specifications To calculate the damage at a 35% reduction in G*Sin*δ*, the value obtained from Equation (3) is used:

(3) $$D_{f}=(0.35)({\frac{C_{0}}{C_{1}})}^{\frac{1}{C_{2}}}$$

where C is a regression coefficient. The binder performance parameters were calculated using Equation (4) as follows:

(4) $$A_{35}=\frac{f({D_{f})}^{K}}{k{\left(\pi I_{D}C_{1}C_{2}\right)}^{\alpha }}$$

where:

*f* = loading frequency (10 Hz)

*k* = $1+(1-C_{2})\alpha$

*B* = 2α. 

Finally, Equation (5) was used to calculate the number of cycles to fatigue.

(5) $$N_{f}=A_{35}(\gamma_{max}{)}^{-B}$$

where *γ_max_* is the maximum expected binder strain for a given pavement structure.

The test was performed on asphalt binder samples of 8 mm in diameter after long-term aging in the pressure aging vessel (PAV). The LAS uses a two-step approach. The first step was to apply a shear frequency sweep to obtain the linear viscoelastic properties. The frequency sweep test was performed at a frequency of 10 Hz, linearly increasing the strain amplitudes from 0.1 to 30% over 3100 loading cycles (10 cycles per second) for a total time of 310 s. The two phases were performed in succession at an intermediate temperature, as specified in the Superpave system [(high PG temperature + low PG temperature)/2], to obtain the asphalt binder’s undamaged and damaged material properties. The test was performed at multiple frequencies for 10 s intervals, whereas, in the current LAS test procedure, the calculation of test results was based on the utilization of the viscoelastic continuum damage (VECD) theory. The VECD theory combines the principles of viscoelasticity and continuum damage mechanics to provide a description of the material behavior. It involves constitutive models that represent the stress–strain relationship in the material, to predict how pavements will perform over time in terms of accumulation of damage [56].

An Excel sheet developed by the Modified Asphalt Research Center at the University of Wisconsin-Madison [57] was utilized to carry out the analysis. The number of cycles to fatigue failure (N_f_) was the primary output of the LAS test. 

#### 3.4.5. Bending Beam Rheometer (BBR) Test

The Bending Beam Rheometer (BBR) test ASTM D6648 [58], was used in Superpave to test the Pressure Aging Vessel (PAV)-aged asphalt binder at low temperatures for thermal cracking. The BBR basically subjected an asphalt binder simple beam with dimensions of 6.25 × 12.5 × 127 mm to a constant creep load of 0.981 N (resulting from a 100 g mass) over 240 s (creep test). The BBR test simulated the asphalt binder stiffness after two hours of loading at the minimum HMA pavement design temperature. The BBR test investigated the stiffness and relaxation characteristics of the asphalt binder at low temperatures. From the BBR test, the deformation with time was recorded, and therefore, the beam’s creep stiffness was plotted with time. The measurements of the creep stiffness (S(t)) showed the low-temperature cracking susceptibility of the asphalt binder and were related to the thermal stresses in an HMA pavement due to shrinking. The rate of change in the stiffness with time (the slope of the curve of the stiffness with time or the m-value) at 60 s was also obtained. The m-value indicates the ability of the HMA pavement to relieve stresses at low temperatures. The Superpave specifications require in the BBR test a maximum value of 300 MPa for the creep stiffness and a minimum value of 0.300 for the m-value. The low testing temperature in the BBR was used to identify the low-temperature performance in the Superpave performance grading (PG) system. If a binder met the criteria for a certain testing temperature, this temperature was shifted down in the grading system by (10 degrees).

#### 3.4.6. Rolling Thin-Film Oven (RTFO) Test

The Rolling Thin-Film Oven (RTFO) test, ASTM D2872] [59], simulated the short-term aging in asphalt binders during the mixing and laydown of HMA. Standard bottles of asphalt binder (35 g in each) were placed in a rack in the RTFO maintained at a temperature of 163 °C and subjected to air flow at a rate of 4 L/min. The rack rotated at a specified rate in a vertical plane. The test lasted for 85 min from the time the samples were placed in the oven (it was assumed that a period of 10 min was sufficient to allow the temperature to stabilize back to 163 °C after opening the oven door to place the samples).

#### 3.4.7. Pressure Aging Vessel (PAV) Test

**Figure 2:**
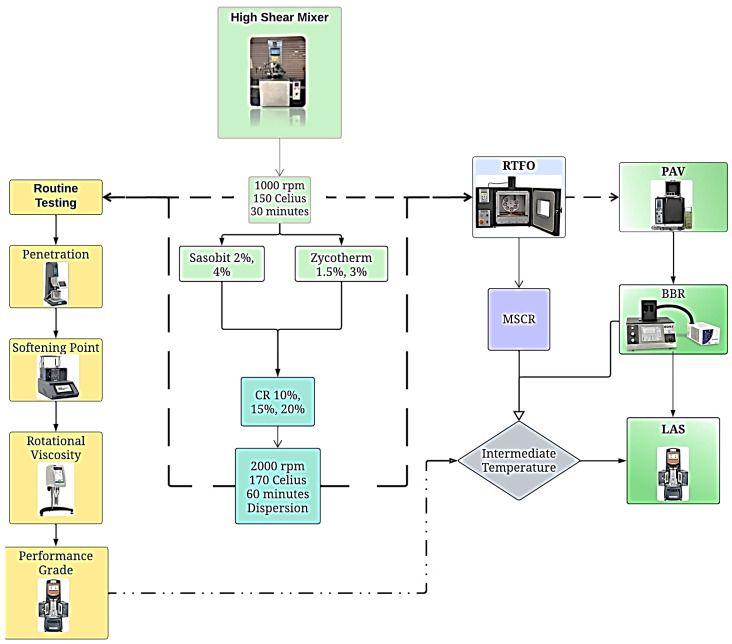
Flowchart summarizing the testing methods in the study.

Long-term aging was simulated using the Pressure Aging Vessel (PAV), manufactured by ATS, Shreveport, LA, USA in accordance with [ASTM D6521] [60]. The PAV test simulated the long-term aging that occurs in asphalt binders during the service life of the HMA pavement. The RTFO residue was poured into PAV standard pans at 50 g each and the samples were placed inside the PAV. The PAV is an oven-pressure vessel combination that takes RTFO-aged samples and exposes them to a high air pressure (2070 kPa = 300 psi) and high temperature (90 °C = 195 °F, 100 °C = 212 °F, or 110 °C = 230 °F) depending upon expected climatic conditions for 20 h. In this study, the temperature used was 110 °C due to the very hot climate in the study area. A flowchart summarizing the test procedures is shown in Figure 2.

## 4. Analysis of Results and Discussion

### 4.1. Warm Mix Asphalt Binders (Without Crumb Rubber)

**Figure 3:**
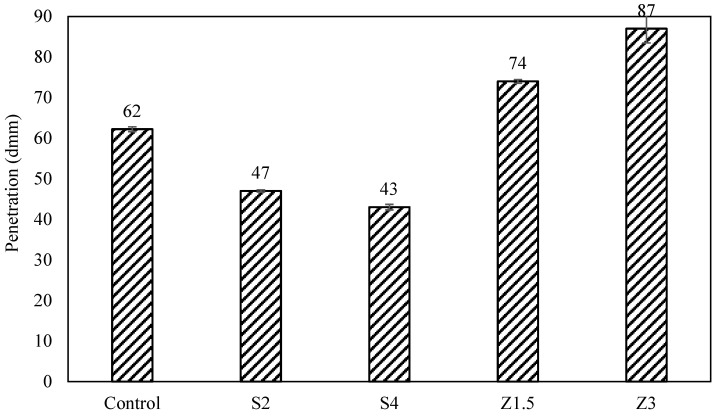
Penetration values for warm mix asphalt.

Sasobit and Zycotherm’s impacts on the penetration, softening point, and viscosity of the asphalt binder were tested. The penetration results presented in Figure 3 show a 24% and 31% decrease for S2 and S4, respectively. Zycotherm, on the other hand, showed increases in penetration of 19% and 40%. This indicates an increase in the stiffness of the Sasobit-modified asphalt binder and a decrease in the stiffness of the Zycotherm-modified asphalt binder. 

**Figure 4:**
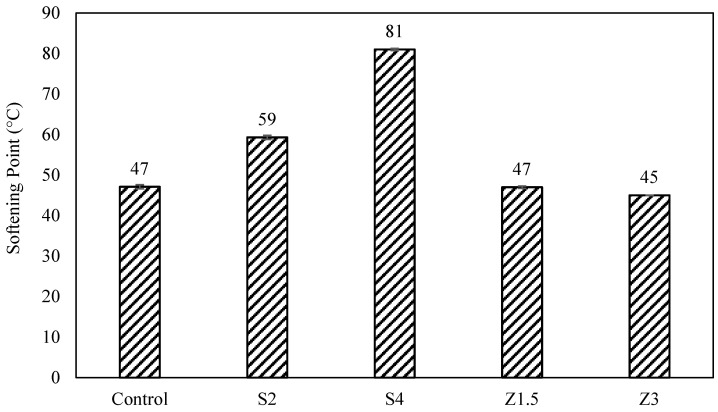
Softening point results for warm mix asphalt.

The softening point values, Figure 4, showed a similar trend to the penetration. Sasobit significantly increased the softening point temperature by 26% and 72% for S2 and S4, respectively. Z1.5 did not change the softening point, while Z3 decreased the softening point slightly. The penetration decreased and the softening point increased when using Sasobit. This was due to the crystallization of Sasobit in the asphalt and the long hydrocarbon chains of Sasobit, thus increasing the stiffness and stability at intermediate temperatures. Previous studies have shown the ability of wax-based warm mix additives to decrease the penetration and increase the softening point, while chemical warm mix additives have shown various impact on the physical properties of asphalt binders, depending on the nature of the chemical additive used [11].

**Figure 5:**
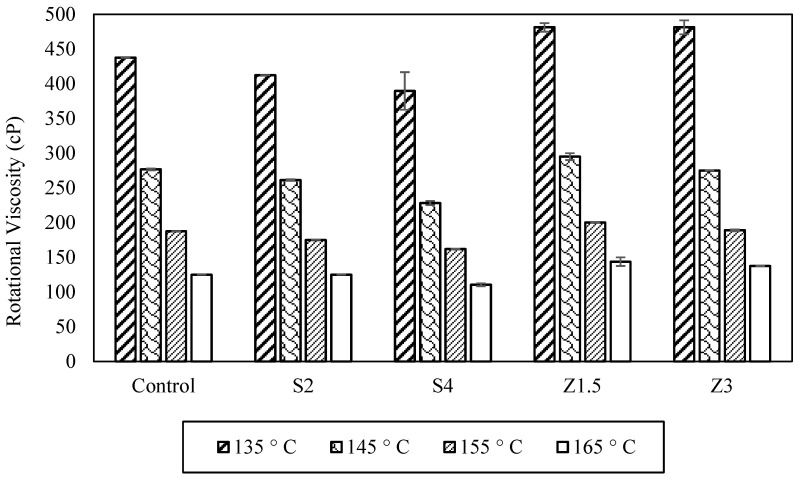
Rotational viscosity of the modified asphalt binders.

The rotational viscosity of the asphalt binders, Figure 5, at 135 °C decreased by 6% and 17% for S2 and S4, respectively. Z1.5 and Z3 recorded slightly increased viscosities by 9% and 8%, respectively. At 165 °C, S2 maintained the viscosity while S4 decreased the RV by 12%. Z1.5 and Z3 increased the viscosity by 15% and 10%, respectively. Viscosity temperature charts were used to calculate the mixing and compaction temperatures, as shown in Table 5. Based on the calculations, S2 and S4 lowered the mixing and compaction temperatures by 1 °C and 3 °C, respectively. Meanwhile, Z1.5 increased the mixing temperature by 3 °C and the compaction by 2 °C. Increasing the Zycotherm content to 3% increased the mixing temperature by 1 °C. This slight increase with Zycotherm was due to the chemical change that the asphalt binder underwent using chemical additives, and another reason could be the high dosage used in this study. The decrease in the viscosity when Sasobit was added was due to the melting of the Sasobit. Sasobit decreased the rotational viscosity of the asphalt binder in its liquid form due to the long hydrocarbon chains in the mix. The rotational viscosity results are supported by findings of previous studies conducted on warm mix additives [14,17,47].

**Figure 6:**
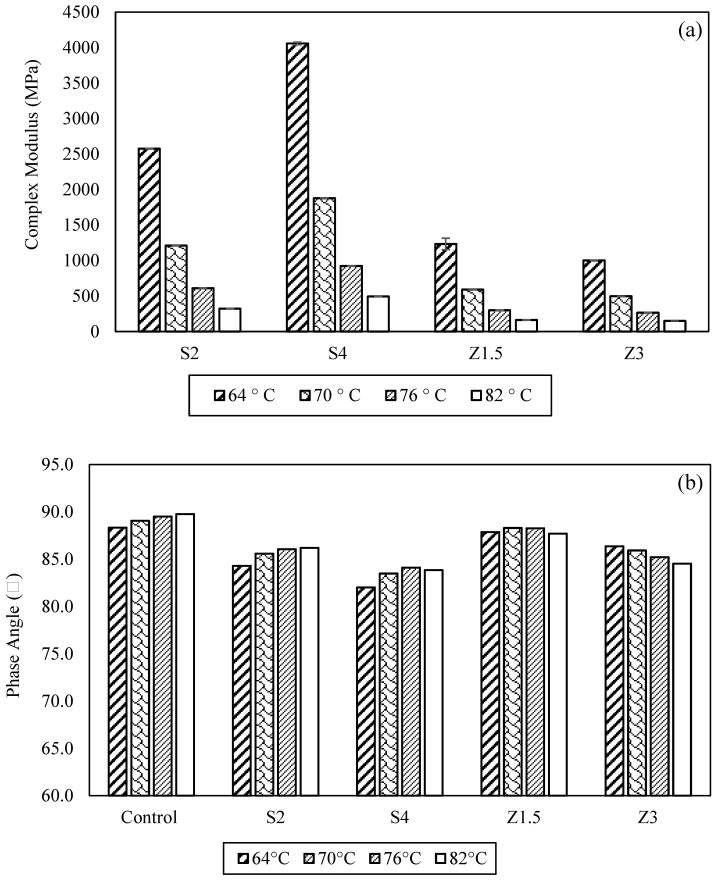

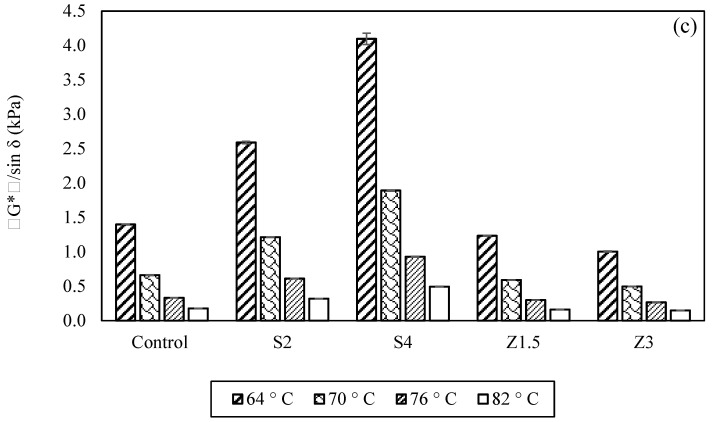
Dynamic shear test results, (**a**) complex shear modulus, (**b**) phase angle, and (**c**) rutting parameter.

At 64 °C, the Sasobit-modified asphalt binders exhibited a doubling and quadrupling of the complex shear modulus value (|G*|) and stiffness for S2 and S4, respectively (Figure 6, Complex Shear Modulus, Phase Angle, and Rutting Parameter Results). In contrast, the Zycotherm modifier led to an 11% and 28% reduction in |G*| for Z1.5 and Z3, respectively.

In terms of the phase angle (δ), which indicates the viscoelastic behavior of asphalt binders, all the warm mix asphalt binders demonstrated a reduction in δ, indicating that the binders were becoming more elastic. The most pronounced decrease in the phase angle was observed in the S2 and S4 asphalt binders, as shown in Figure 6. 

The S2 and S4 asphalt binders exhibited an increase in their Performance Grade (PG) from PG 64 to PG 70. At 64 °C, S2 and S4 displayed |G*|/sin*δ* values that were 46% and 66% higher, respectively, indicating a superior resistance to rutting, as depicted in Figure 6. On the other hand, Zycotherm did not enhance the PG grade, but reduced |G*|/sin*δ* by 11% and 28% for Z1.5 and Z3, respectively, compared to the control asphalt binder.

The Sasobit-modified asphalt binders (S2 and S4) demonstrated a higher resistance to rutting deformation at 64 °C, attributed to their high |G*| and low phase angle. These findings agree with earlier studies that utilized Sasobit at 4% to enhance high-temperature performance, owing to the crystallization of Sasobit in the asphalt binder below its melting temperature [7,9,14,16]. Similarly, Zycotherm showed a minimal impact on the asphalt binder’s rheological properties, confirming previous studies [29,30]. 

The BBR creep stiffness measurements serve as an indicator of the asphalt binder’s susceptibility to low-temperature cracking. Lower stiffness values are associated with a high thermal cracking (low temperature) resistance. As the CR content increased, both S2CR asphalt binders exhibited a decrease in their creep stiffness compared to the control asphalt binder S2 (CR = 0%). However, the S4CR asphalt binders displayed a slightly different trend, with creep stiffness values approximately similar to the control asphalt binder S4 (CR = 0%).

In contrast, the S2CR10, S2CR15, and S2CR20 binders showed significantly lower stiffnesses by 11%, 43%, and 43%, respectively, compared to the control binder S2. For the S4CR10, S4CR15, and S4CR20 binders, their creep stiffness values were similar to the control binder S4 at −6 °C.

Regarding the Z1.5CR and Z3CR asphalt binders, notable creep stiffness reductions were observed compared to the control binders, the Z1.5 and Z3 asphalt binders. Specifically, the Z3CR10, Z3CR15, and Z3CR20 asphalt binders displayed lower creep stiffness values, reduced by 13%, 11%, and 12%, respectively, compared to the Z3 asphalt binder. On the other hand, the Z1.5CR asphalt binder showed a decrease in creep stiffness of 13% at a CR content of 10%. The lack of enhancement in creep stiffness observed in Z1.5CR15 and Z1.5CR20 indicates that the 10% CR content was likely the optimal value for achieving the desirable creep stiffness in this group of CR-modified WMA asphalt binders. The Z1.5CR binder exhibited results beyond the linear viscoelastic range, which is more realistic in representing actual field situations where the binder is subjected to traffic loads. The MSCR test results presented in Table 6 reveal that the addition of Sasobit at 64 °C with a stress level of 3.2 kPa improved the creep recovery significantly. Specifically, with a 2% Sasobit content, the creep recovery increased from 0.4% to 1.77%, and with a 4% Sasobit content, the creep recovery increased to 5.11%. On the other hand, the use of Zycotherm did not show any enhancement in the recovery. S2 did not meet the MSCR requirements for PG70, while S4, with its improved creep recovery performance, successfully fulfilled the MSCR criteria.

The low-temperature performance grade (PG) was determined using the Bending Beam Rheometer (BBR) test results. The m-value results obtained from this test offer valuable insights into the resistance to thermal cracking, as illustrated in Table 7. Adding Sasobit to the asphalt binders reduced the low-temperature PG, reaching −16 and −10 for S2 and S4, respectively. Z1.5 exhibited a low-temperature performance similar to S2 (−16), while Z3 demonstrated comparable results to the control asphalt binder, with a PG of −22. The findings indicated a decrease in creep stiffness with Sasobit at both 2% and 4% contents, whereas Zycotherm increased the creep stiffness from the BBR test. Z3 exhibited a creep stiffness similar to that of the control asphalt binder.

The performance grade classification of each asphalt binder was established by considering all the results obtained from the PG, MSCR, and BBR tests, as presented in Table 8.

The BBR results supported the findings of previous studies, confirming that Sasobit enhances high-temperature performance due to increased stiffness, while renders the asphalt binder more susceptible to low-temperature cracking [22]. 

The intermediate temperatures were determined using the high and low PG temperatures for the asphalt binders. The intermediate temperature can be calculated as the sum of the high and low PG grades, divided by two, with four added to the result. The viscoelastic continuum-damage (VECD) theory was employed to analyze the data. The VECD theory combines the principles of viscoelasticity and continuum damage mechanics to provide description of material behavior. It involves constitutive models that represent the stress–strain relationship in the material, to predict how pavements will perform over time in terms of accumulation of damage [55].

**Figure 7:**
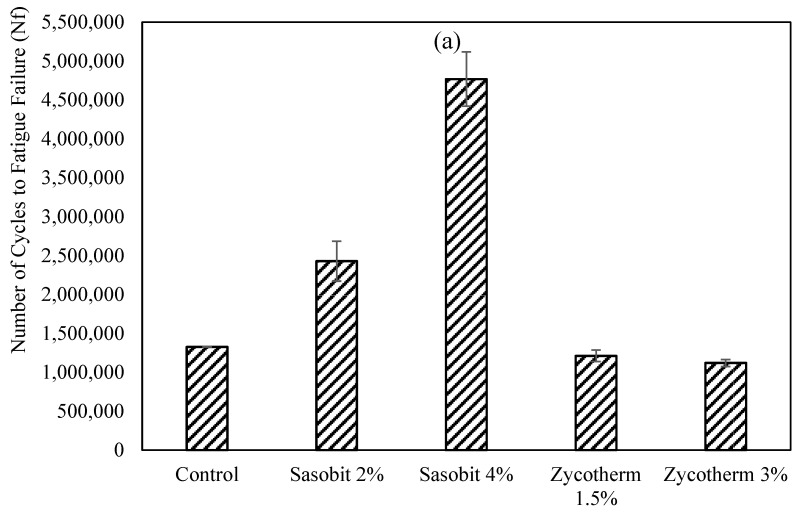

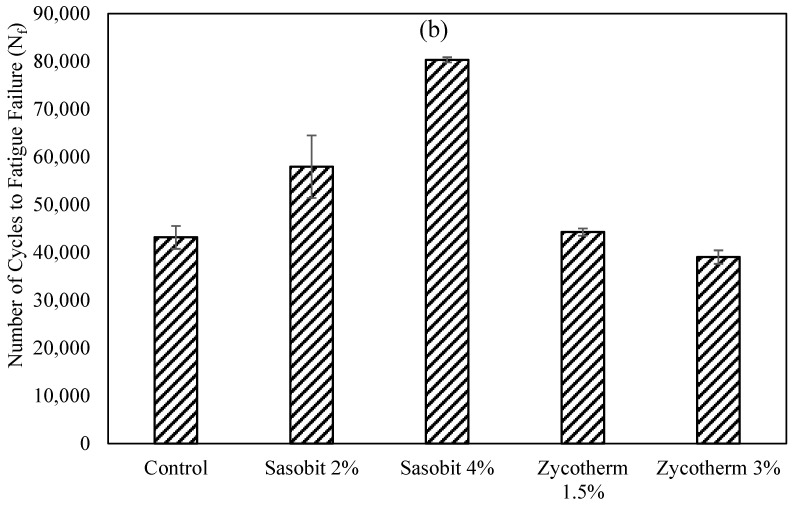
LAS test results at 28 °C, (**a**) at 2.5% strain level and (**b**) at 5.0% strain level.

Figure 7a illustrates the influences of various additives on the number of cycles to fatigue failure at a 2.5% strain level. S2 showed a remarkable increase of 83% in the number of cycles to fatigue failure, while S4 increased it by more than threefold, resulting in a significant improvement in fatigue life. On the other hand, the Z1.5 and Z3 binders did not show any enhancement in the number of cycles to fatigue failure. Incorporating Sasobit reduced the aging effects of the asphalt binders, mainly by lowering the compaction and mixing temperatures, leading to an increase in fatigue life.

At 5% strain levels (Figure 7b), again, the influence of Sasobit was more pronounced than that of Zycotherm. S2, S4, and Z1.5 increased the number of cycles to fatigue failure by 34%, 86%, and 3%, respectively, while Z3 decreased it by 10%. Previous studies have indicated that Sasobit’s effectiveness in reducing aging is attributed to lower M&C (Mixing and Compaction) temperatures of the asphalt binder, resulting in a reduced stiffness, whereas Zycotherm has a minimal effect [7,9,30].

### 4.2. Crumb Rubber-Modified Warm Mix Asphalt Binders

Traditional tests such as penetration, softening point, and rotational viscosity were performed to evaluate the influence of crumb rubber on warm mix asphalt binders. Advanced characterization tests were also conducted to further understand the impact of crumb rubber on the binders. Each rubber content was mixed with four warm mix additives.

**Figure 8:**
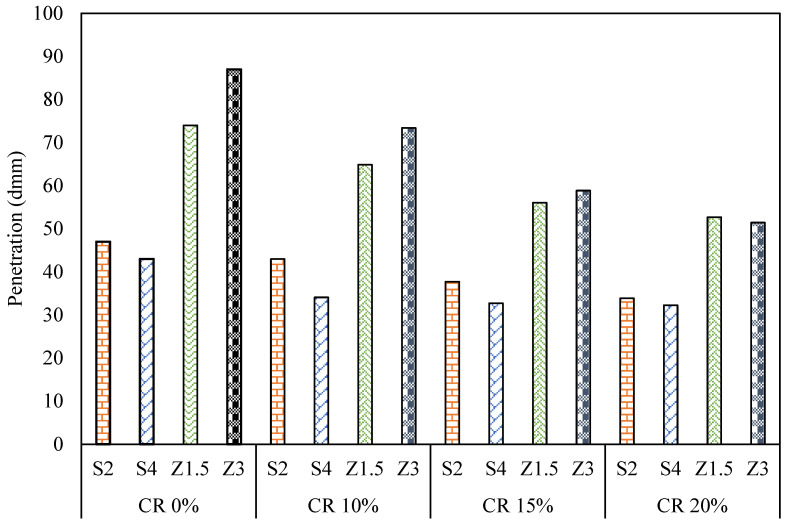
Penetration results for CR/WMA binders.

The penetration test results for CR demonstrated a consistent reduction in penetration values, as depicted in Figure 8. The findings indicated that increasing the CR content decreased the penetration values of the asphalt binder. Specifically, with an increase in CR content from 10% to 15% to 20%, the penetration of S2CR decreased by 9%, 20%, and 28, respectively. Similarly, the penetration of S4CR decreased by 21%, 24%, and 25%. In contrast, Z1.5CR and Z3CR experienced penetration reductions of 12%, 24%, and 29% and 16%, 32%, and 41% respectively. This shows that the reduction in penetration for Sasobit binders was higher than the reduction observed for Zycotherm binders.

**Figure 9:**
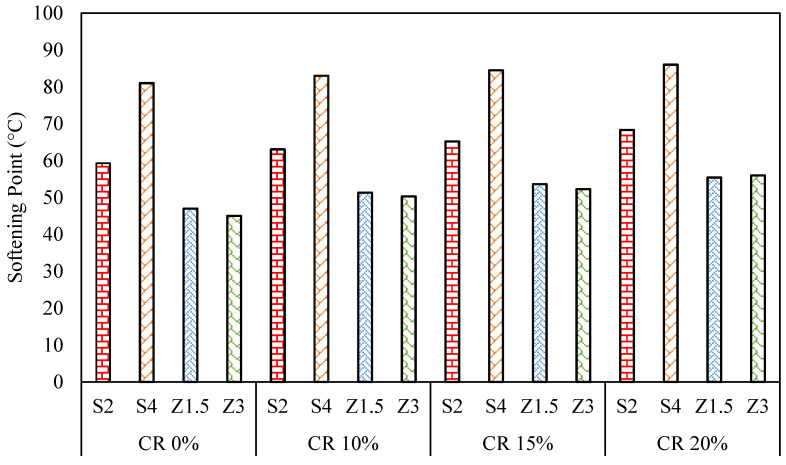
Softening point results for CR/WMA binders.

As depicted in Figure 9, the softening point results reveal an overall increase in the CR-modified WMA binders compared to the WMA binders. Specifically, the Sasobit blends demonstrated significantly higher softening point temperatures than the Zycotherm blends.

Increasing the CR content increased the softening points for the Sasobit and Zycotherm asphalt binders, however, this increase was more observable in the Sasobit binders than the Zycotherm binders. At CR contents of 10, 15, and 20%, the softening point exhibited percentage increases of 6, 10, and 15% for S2CR, respectively, and for S4CR, the percentage increases were 3, 5, and 7%, respectively. However, for Z1.5CR, the softening point increased by 10, 15, and 19% at CR contents of 10, 15, and 20%, correspondingly, and for Z3CR, the softening point experienced percentage increases of 11, 15, and 23% at CR contents of 10, 15, and 20%, respectively. 

This observation indicates that the rise in the softening point was directly linked to the increase in the crumb rubber content. Notably, the substantial increase in the softening point temperature was because the 20% CR-modified mix was considered to be rubberized, as it contained a high proportion of crumb rubber. The presence of rubber particles increased the friction with the balls and the rings when the modified binder was traveling down from its original position.

Both WMA binders, through their ability to absorb the CR particles, enhanced the compatibility between the crumb rubber and the asphalt binders, thus promoting the stiffness of the asphalt binder [7,9]. This explains the positive impact on the softening point results observed in the study.

The viscosity of asphalt binders is crucial to the mixability and workability of asphalt binders with aggregates. The rotational viscosity results in Figure 9 indicate that adding CR to the WMA binders significantly increased the viscosity at temperatures of 135 °C, 145 °C, 155 °C, and 165 °C.

At the four temperatures, the increase in the CR content increased the rotational viscosity for the Sasobit and Zycotherm asphalt binders, with the Sasobit binders having a lower viscosity at all temperatures. At the standard temperature (135 °C), the rotational viscosity for S2CR compared to the control binder (S2, CR = 0%) increased by 2, 4, and 7 times at CR contents of 10, 15, and 20%, respectively. For S4CR, similarly, the rotational viscosity experienced increases of 2, 3, and 4 times compared to the control binder (S4, CR = 0%) at CR contents of 10, 15, and 20%, respectively.

A comparable trend emerged with the Zycotherm asphalt binders. In the case of Z1.5CR, the rotational viscosity was enhanced 2, 3, and 5 times at CR contents of 10, 15, and 20%, respectively. Meanwhile, for Z3CR, the rotational viscosity demonstrated corresponding rises of 2, 3, and 6 times at CR contents of 10, 15, and 20%, respectively.

The mixing and compaction (M&C) temperatures, as shown in Table 9, were calculated based on the penetration, softening point, and rotational viscosity results. The inclusion of CR led to a substantial increase in the M&C temperature ranges. Compared to results in other studies [36], increasing the CR content increased the mixing temperatures by 15%, 24%, and 33% at CR contents of 10%, 15%, and 20%, respectively, for the S2CR asphalt binder. For the S4CR binder, the percentages were 13%, 22%, and 27%, respectively. On the other hand, the Z1.5CR and Z3CR binders encountered increases in mixing temperature of 10%, 21%, and 29% and 9%, 25%, and 33% at CR contents of 10%, 15%, and 20%, respectively. Comparing the mixing temperatures between the additives, for 10% CRM, Zycotherm was found to have a much lower M&C compared to the Sasobit-modified binders. This was also true for a higher CRM content. A higher Sasobit content increased the (M&C) temperatures, while a higher Zycotherm content did not change the temperatures as much.

Despite the high temperatures, adding CR to the WMA binders resulted in a lower viscosity than the CR-modified hot mix asphalt binders, mainly due to the melting of the warm additives at high temperatures. The interaction between the warm mix additives and crumb rubber reduced the activation energy of the CR mix particles, leading to a viscosity reduction [33,34].

**Figure 10:**
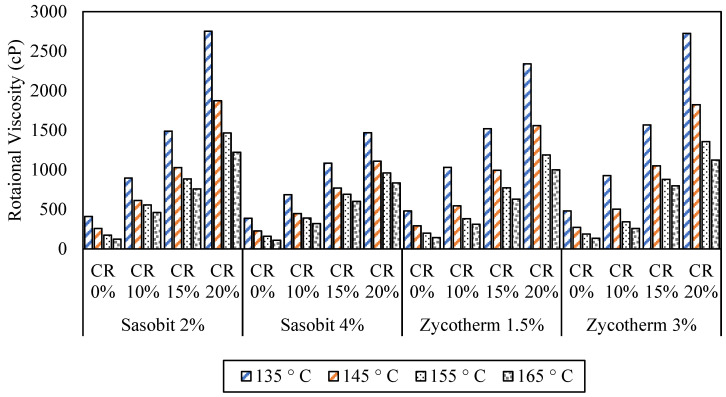
Rotational viscosity results for CR/WMA binders.

The rheological properties presented in Figure 10 for the CR-modified WMA binders demonstrate a substantial expected enhancement compared to the WMA binders. The complex shear modulus, phase angle, and rutting parameter showed significant improvements. Incorporating the CR and WMA binders significantly boosted the G* value for both the Sasobit and Zycotherm binders. Specifically, S2CR10, S2CR15, and S2CR20 displayed two, three, and four times, respectively, higher G* values than the S2 asphalt binder (control binder) at all temperatures. Similarly, S4CR10, S4CR15, and S4CR20 approximately doubled the G* value of the S4 asphalt binder (control binder). 

In the case of Zycotherm, the introduction of CR to Z1.5CR10, Z1.5CR15, and Z1.5CR20 displayed two, three, and four times increases in the G* value, respectively. As for the Z3CR10, Z3CR15, and Z3CR20 binders, they demonstrated a twofold, threefold, and fivefold increase in the G* value, respectively. However, when compared to the Sasobit binders, the Zycotherm binders exhibited significantly lower G* values. Moreover, increasing the Zycotherm content in the CR-modified WMA binders led to a decrease in the G* value. 

Regarding the phase angle results, generally, the inclusion of CR slightly decreased the lag response (phase angle), especially at a 10% CR content, indicating a more elastic response. Nonetheless, in the case of asphalt binders containing 15% and 20% CR, an increase in CR content led to a partial reduction in the phase angle. Specifically, for S2CR and S4CR, the decreases at 64 °C amounted to 7% and 11%, and 7% and 7% at CR contents of 15% and 20%, respectively. As for Z1.5CR and Z3CR, these percentages were 6% and 7%, and 5% and 7%, respectively, yielding a higher elastic response with a higher Zycotherm content.

**Figure 11:**
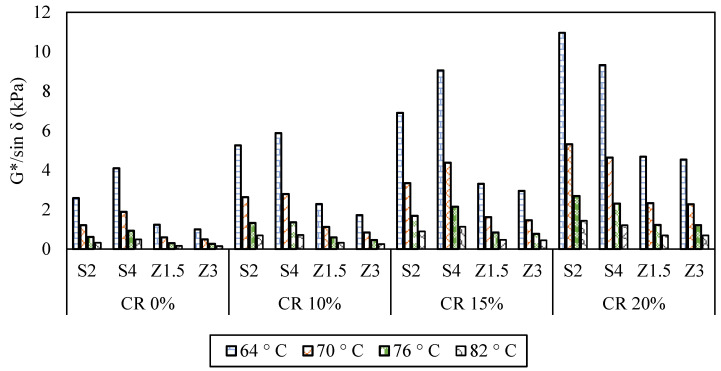
Rutting parameter (G*/sin*δ*) results.

As illustrated in Figure 11, the |G*|/sin*δ* results exhibited a decrease as the temperature rose. However, the addition of CR significantly enhanced the |G*|/sin*δ* values. Specifically, S2CR20 and S4CR20 increased the PG from 64 to 82, indicating an improved rutting resistance of the asphalt binder due to an increased stiffness. Similarly, incorporating CR into the Zycotherm asphalt binders showed a comparable trend in enhancing their high-temperature performance. Compared to Sasobit, Zycotherm achieved a maximum PG of 76 with 20% CR, showcasing its superior rutting performance.

Table 10 presents the MSCR (Multiple-Stress Creep Recovery) test results for all asphalt blends incorporating CR. The influence of CR on the WMA binders was evaluated through the MSCR test at 64 °C. As anticipated, adding CR improved the rutting resistance and creep recovery of the asphalt binder at a stress level of 3.2 kPa.

Compared to the S2 asphalt binder, S2CR10, S2CR15, and S2CR20 demonstrated an enhanced creep recovery of the asphalt binder. While the S4CR10, S4CR15, and S4CR20 binders also exhibited an improved creep recovery, this enhancement was notably lower than that observed in the S2CR binders. Remarkably, the Z1.5CR and Z3CR binders, despite being lower in G* value, showed the most significant improvements in the creep recovery of the asphalt binder.

In comparing the rubberized WMA binders, the S2CR20 binder achieved the highest creep recovery, followed by S4CR20, Z1.5CR20, and Z3CR20. Additionally, an increase in the CR content enhanced the creep recovery for all CR-modified WMA binders. This suggests that an increase in the stiffness of the asphalt binder positively impacted the creep recovery capability of the asphalt binder.

The rutting performance, as indicated by the non-recoverable creep compliance parameter at a stress level of 3.2 kPa (J_nr3.2_), was evaluated for the various modified asphalt binders. In the case of the S2CR10, S2CR15, and S2CR20 binders, the J_nr3.2_ parameter exhibited reductions of 68%, 77%, and 91%, respectively, in comparison to the control binder S2. Similarly, for the S4CR10, S4CR15, and S4CR20 binders, the J_nr3.2_ parameter displayed decreases of 63%, 65%, and 80%, respectively, compared to the control binder S4. As for Z1.5CR10, Z1.5CR15, and Z1.5CR20, the decreases were 70%, 71%, and 88% in the J_nr3.2_ parameter relative to the Z1.5 control binder. Likewise, the Z3CR10, Z3CR15, and Z3CR20 binders experienced reductions of 53%, 75%, and 87%, respectively, in the J_nr3.2_ parameter compared to the Z3 control binder.

In contrast, the S2CR10, S2CR15, and S2CR20 binders showed significantly lower stiffness by 11%, 43%, and 43%, respectively, compared to the S2 control binder. The creep stiffness values of the S4CR10, S4CR15, and S4CR20 binders were similar to the S4 control binder at −6 °C. Regarding the Z1.5CR and Z3CR asphalt binders, notable creep stiffness reductions were observed compared to the control binders, the Z1.5 and Z3 asphalt binders. Specifically, the Z3CR10, Z3CR15, and Z3CR20 asphalt binders displayed lower creep stiffness values, reduced by 13%, 11%, and 12%, respectively, compared to the Z3 asphalt binder. On the other hand, the Z1.5CR asphalt binders showed decreases in creep stiffness. Specifically, the Z3CR10, Z3CR15, and Z3CR20 asphalt binders displayed lower creep stiffness values, reduced by 13%, 11%, and 12%, respectively, compared to the Z3 asphalt binder. On the other hand, the Z1.5CR asphalt binders showed a decrease in creep stiffness of 13% at a CR content of 10%. The lack of enhancement in creep stiffness observed in Z1.5CR15 and Z1.5CR20 indicates that the 10% CR content is likely the optimal value for achieving desirable creep stiffness in this CR-modified WMA asphalt binders group.

The traffic levels can be determined from AASHTO [M332] based on the J_nr3.2_ values in Table 10. Across all CR WMA binders, the pavement’s capacity to endure traffic showed an enhancement as the CR content increased from 0% (control binder) to 10%, 15%, and 20%. To illustrate, considering the S2CR binder, its traffic level improved from standard (S), corresponding to the control binder with CR at 0%, to Extremely Heavy (E) when the CR content reached 20%. A parallel pattern can be observed for the S4CR binder, with its traffic level improving from Heavy (H) to “E”. Conversely, in the cases of the Z1.5CR and Z3CR binders, the traffic levels advanced from “S” to “E” and from “S” to Very heavy (V), respectively.

S2CR20 demonstrated the best rutting resistance, displaying the highest creep recovery and the least non-recoverable creep compliance (J_nr3.2_). An increase in Sasobit content positively impacted the asphalt binder’s high-temperature performance, as the interaction between Sasobit and CR enhanced its rutting performance, in line with previous studies [17,40,42]. Moreover, the use of Sasobit is preferred over chemical warm mix additives [42]. 

**Figure 12:**
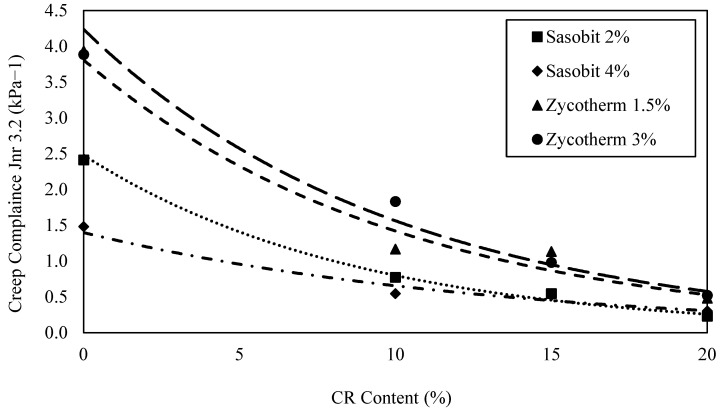
Jnr_3.2_ versus CR content at 64 °C.

**Figure 13:**
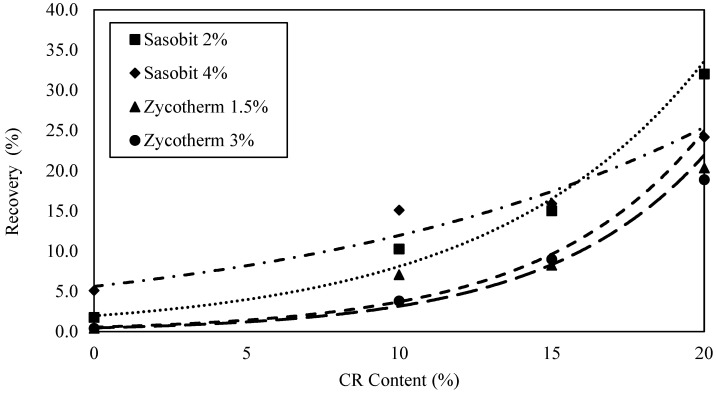
R_3.2_ versus CR content at 64 °C.

The J_nr3.2_ and %R values were graphed against the CR content for all the WMA binders, as presented in Figure 12 and Figure 13. The exponential model was the most suitable in describing the relationship between J_nr3.2_ and %R at 64 °C with the CR content. As the CR content increased, there was an exponential decrease in the J_nr3.2_ value and a simultaneous exponential increase in the creep recovery (%R). This indicates that adding CR significantly improved the rutting resistance for the WMA binders.

Nevertheless, no clear trend emerged when the J_nr3.2_ or %R values at the high PG temperature were plotted against the CR content. This discrepancy can be attributed to the fact that these values were obtained at different temperatures for each asphalt binder based on its high PG temperature. The addition of the rubber enhanced the binder’s resistance to creep due to the internal friction of the solid rubber particles. The recovery of the rubber-modified warm mix binders was improved by the gel-like behavior of the rubber particles interacting with the WMA binders.

Table 11 presents the BBR test results, revealing appealing observations. The addition of CR to the S2 asphalt binder did not affect the low PG of the asphalt binder, but when added to the S4 asphalt binder, it reduced the low PG by 6 °C. The S2CR binders did not show any improvement (increase) in the m-value. However, S4CR binders showed enhancements in the m-value, resulting in an improved low PG grade. Conversely, the Z1.5CR binders had higher m-values, particularly with more CR content, leading to lower PG grades. The most significant enhancement was seen in the Z3CR binders, with the m-value improvement resulting in enhanced low PG grades of −28 and −34 at 15% and 20% CR contents, respectively.

Regarding the Z1.5CR asphalt binders, Z1.5CR10 displayed the same low PG as Z1.5, whereas Z1.5CR15 and Z1.5CR20 exhibited an improved low PG of −22. The most significant improvement in thermal cracking resistance was observed in the Z3CR asphalt binder group. Z3CR15 and Z3CR20 demonstrated low PG values of −28 and −34, respectively, indicating considerable enhancements in their performance.

BBR creep stiffness measurements serve as an indicator of an asphalt binder’s susceptibility to low-temperature cracking. Lower stiffness values are associated with a high thermal cracking (low-temperature) resistance. As the CR content increased, both S2CR asphalt binders exhibited a decrease in their creep stiffness compared to the S2 control asphalt binder (CR = 0%). However, the S4CR asphalt binders displayed a slightly different trend, with creep stiffness values approximately similar to the S4 control asphalt binder (CR = 0%).

In contrast, the S2CR10, S2CR15, and S2CR20 binders showed significantly lower stiffnesses by 11%, 43%, and 43%, respectively, compared to the S2 control binder. For the S4CR10, S4CR15, and S4CR20 binders, their creep stiffness values were similar to the S4 control binder at −6 °C.

Regarding the Z1.5CR and Z3CR asphalt binders, notable creep stiffness reductions were observed compared to the control binders, the Z1.5 and Z3 asphalt binders. Specifically, the Z3CR10, Z3CR15, and Z3CR20 asphalt binders displayed lower creep stiffness values, reduced by 13%, 11%, and 12%, respectively, compared to the Z3 asphalt binder. On the other hand, the Z1.5CR asphalt binders showed a decrease in creep stiffness of 13% at a CR content of 10%. The lack of enhancement in creep stiffness observed in Z1.5CR15 and Z1.5CR20 indicates that the 10% CR content was likely the optimal value for achieving the desirable creep stiffness in this group of CR-modified WMA asphalt binders (Z1.5CR).

**Figure 14:**
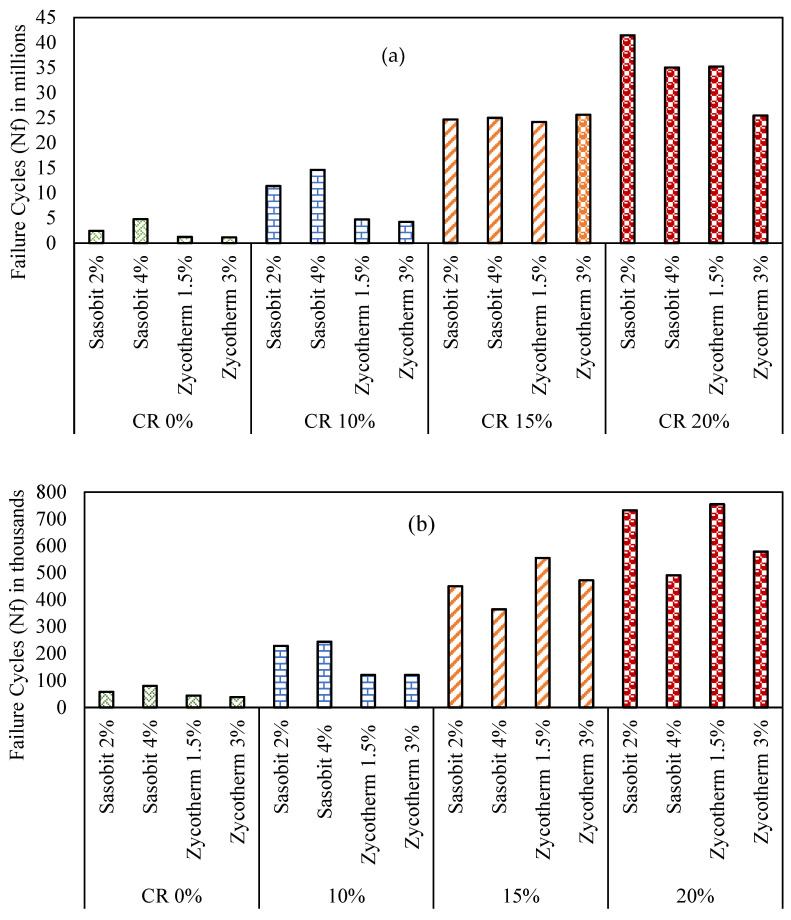
LAS test results (**a**) at 2.5% strain level and (**b**) at 5.0% strain level.

The LAS testing was conducted at 28 °C under 2.5% and 5% strain to evaluate the fatigue performances of the binders, and the results are presented in Figure 14, LAS Test Results. It is clear that the increase in the CR content in the modified WMA binders led to an increased number of cycles to fatigue failure, thus improving the fatigue life. Remarkably, among all the modified binders, S2CR20, followed by Z1.5CR20, demonstrated the longest fatigue life (N_f_) at the 2.5% strain level. And at the 5% strain level, Z1.5CR20, followed by S2CR20, showed the highest fatigue life (N_f_). This indicates an improved fatigue performance at a 20% CR content.

At the 2.5% strain level, the increases in the number of cycles to fatigue failure for the S2CR binders were 5%, 10%, and 17%, at CR contents of 10%, 15%, and 20%, respectively. The same percentages were 3%, 5%, and 7% for the S4CR binders. On the other hand, the Z1.5CR and Z3CR binders experienced increases of 4%, 20%, and 29% and 4%, 23%, and 23%, respectively.

At the strain level of 2.5%, the increases in the cycles to fatigue failure for the S2CR binders were 5%, 10%, and 17%, corresponding to CR contents of 10%, 15%, and 20%, respectively. The corresponding improvements were 3%, 5%, and 7% for the S4CR binders. Conversely, the Z1.5CR and Z3CR binders displayed higher enhancements of 4%, 20%, and 29% and 4%, 23%, and 23%, respectively.

Conversely, when considering a strain level of 5%, the fatigue life of the S2CR and S4CR binders indicated increases of 4%, 8%, and 13% and 3%, 5%, and 6%, respectively, at CR contents of 10%, 15%, and 20%. Meanwhile, the Z1.5CR and Z3CR binders exhibited better improvements of 3%, 13%, and 17% and 3%, 12%, and 15%, respectively, at CR contents of 10%, 15%, and 20%.

Previous studies have suggested the use of Sasobit to enhance the fatigue life of asphalt binders by reducing oxidation and volatilization, thereby mitigating the aging impact of the asphalt binder [22,30]. However, this study demonstrates a notable improvement in fatigue life by employing Zycotherm at 1.5% and incorporating 20% crumb rubber.

## 5. Limitations

It is important to note that the test results presented in this research work are limited to the mixing procedures, testing conditions, and type and concentration of the materials. Therefore, changing any of those inputs may affect the outputs.

## 6. Conclusions

This paper investigated the effect of adding two commercial warm mix additives, namely, Sasobit and Zycotherm, to a control asphalt binder. Crum rubber was then introduced to the warm mix binders and its effect was studied.

### 6.1. Effect of Warm Mix Modifiers

Upon investigating the effects of Sasobit and Zycotherm modifications and analyzing the results of the warm mix asphalt (WMA) binders’ tests, the following conclusions can be drawn:

- Sasobit reduced the penetration and increased the softening point significantly for S2 and S4. In contrast, Z1.5 and Z3 resulted in higher penetration values without causing a significant impact on the softening point.
- The Sasobit-modified asphalt binders showed a better resistance to deformation at high temperatures due to a high |G*| and low phase angle, while Zycotherm showed mixed effects.
- The addition of Sasobit decreased the low-temperature Performance Grade (PG) of the asphalt binders, reaching −16 and −10 for S2 and S4, respectively. Z1.5 exhibited similar low-temperature behavior to S2, whereas Z3 had comparable results to the control asphalt binder, with a PG of −22.
- At a 2.5% strain, S2 saw an 83% increase and S4 more than tripled its cycles to fatigue. At a 5% strain, S2, S4, and Z1.5 increased fatigue life by 34%, 86%, and 3%, respectively, while Z3 decreased it by 10%. The best enhancements were with 4% Sasobit and 1.5% Zycotherm additions.

### 6.2. Effect of Crum Rubber on Warm Mix Binders

Based on the analysis of the results regarding CR-modified warm mix asphalt (WMA) binders, the following conclusions can be inferred:

- The addition of CRM decreased the penetration and viscosity while increasing the softening point. Compared to hot mix CRMB, Sasobit and Zycotherm proved to be effective in reducing the mixing and compaction temperatures, with 3% Zycotherm reducing the mixing and compaction temperatures substantially. The penetration, softening point, and viscosity results align with the rheological findings. The increased physical stiffness increased the G*/Sin.
- All CR-modified WMA binders experienced a decrease in the J_nr3.2_ parameter, indicating an enhanced rutting resistance. These reductions ranged from 63% to 91% for the CR-modified Sasobit binders, and from 53% to 88% for the CR-modified Zycotherm binders, with the most substantial improvements observed in the S2CR20 and Z1.5CR20 asphalt binders. The reduction varied based on the CR content and the warm mix additive content.
- The S2CR10, S2CR15, and S2CR20 binders exhibited lower creep stiffnessed for low-temperature cracking by 11%, 43%, and 43%, respectively, compared to the control binder (S2), while the S4CR10, S4CR15, and S4CR20 binders did not exhibit a notable decrease in their creep stiffness values compared to the control binder (S4).
- Asphalt binders with a 10% CR content showed a reduced creep stiffness and improved low-temperature performance. At 15% and 20% CR levels, the low-temperature PG values notably improved.
- Increasing the CR content in the modified WMA binders led to an enhanced fatigue life. Remarkably, among the Sasobit binders, S2CR20, and among Zycotherm binders, Z1.5CR20, demonstrated the longest fatigue lives at both strain levels.

In summary, and based on this study’s comprehensive findings and analysis, the crumb-rubber-modified Zycotherm WMA binder (Z1.5CR20) is suitable for colder climates. In contrast, the crumb-rubber-modified Sasobit WMA binder (S2CR20) is ideal for hot-climate applications. Future research works should take the testing to the mixtures level and investigate the effect of the crumb rubber WMA on the performance of asphalt mixtures, as well as the rheological correlation between thermal and fatigue cracking.

## Figures and Tables

**Table 1:** Asphalt binder properties.

Property	Result	Standard Test Method
Penetration (mm)	63.0	ASTM D5 [50]
Softening Point (°C)	46.6	ASTM D36 [51]
Rotational Viscosity at 135 °C (Pa·s)	4.38	ASTM D4402 [52]
G*/sin δ at 64 °C (kPa)	1.40	ASTM D6373 [53]

**Table 2:** General properties of the warm mix additives.

Property	Sasobit	Zycotherm
Form	Solid Spheres	Oily liquid
Color	Creamy white	Transparent
Odor	Odorless	Strong chemical odor
Melting Point (°C)	100 to 115 [11]	<0.0 °C
Flash Point (°C)	--	80
Density (g/cm^3^)	0.9	1.01
Viscosity at 135 °C	41.25 cP [22]	1~5
Chemical composition	Aliphatic Polymethelyne hydrocarbon	Hydroxyalkyl-alkoxy-alkyl silyl compounds, benzyl alcohol, and ethylene glycol

**Table 3:** Sieve analysis of crumb rubber.

Diameter (mm)	% Finer
0.840	100
0.600	100
0.420	99.9
0.300	82.8
0.180	35.7
0.150	12.5
0.075	6.1

**Table 4:** Asphalt binders abbreviations.

Warm Mix Additive and Content (%)	Crum Rubber Content (%)	Abbreviation
None	0	Control
Sasobit 2.0%	0	S2
Sasobit 2.0%	10	S2R10
Sasobit 2.0%	15	S2R15
Sasobit 2.0%	20	S2R20
Sasobit 4.0%	0	S4
Sasobit 4.0%	10	S4R10
Sasobit 4.0%	15	S4R15
Sasobit 4.0%	20	S4R20
Zycotherm 1.5%	0	Z1.5
Zycotherm 1.5%	10	Z1.5R10
Zycotherm 1.5%	15	Z1.5R15
Zycotherm 1.5%	20	Z1.5R20
Zycotherm 3.0%	0	Z3
Zycotherm 3.0%	10	Z3R10
Zycotherm 3.0%	15	Z3R15
Zycotherm 3.0%	20	Z3R20

**Table 5:** Mixing and compaction temperatures.

Asphalt Binders	Mixing Temperature Range		Compaction Temperature Range	
	Lower	Upper	Lower	Upper
Control	154	160	138	142
S2	153	160	137	140
S4	151	157	135	139
Z1.5	157	163	140	144
Z3	155	162	139	143

**Table 6:** MSCR test results for WMA binders.

Asphalt Binder/WMA	Temperature (°C)	R_3.2_ (%)	Jnr_3.2_ (kPa^−1^)
Control	64	0.40	2.41
S2	64	1.77	2.41
S4	64	5.11	1.48
S4	70	1.24	4.29
Z1.5	64	0.41	3.93
Z3	64	0.41	3.89

**Table 7:** BBR test results for WMA Binders.

Asphalt Binder/WMA	Testing Temperature (°C)	m-Value	Stiffness (MPa)
Control	−12	0.33	216.93
S2	−6	0.33	125.97
S4	0	0.33	81.41
Z1.5	−6	0.43	99.79
Z3	−12	0.31	207.42

**Table 8:** WMA asphalt binders performance grade.

Asphalt Binder/WMA	High PG	Traffic Level	Low PG	Superpave PG
Control	64	S	−22	64(S) −22
S2	64	S	−16	64(S) −16
S4	70	S	−10	70(S) −10
Z1.5	64	S	−16	64(S) −16
Z3	64	S	−22	64(S) −22

**Table 9:** Mixing and compaction temperatures results.

CR-WMA Binder	Mixing Temperature Range		Compaction Temperature Range	
	Lower	Upper	Lower	Upper
S2CR10	177	184	161	165
S2CR15	190	198	173	177
S2CR20	205	212	185	190
S4CR10	170	176	153	157
S4CR15	184	190	167	171
S4CR20	192	199	174	179
Z1.5CR10	173	180	156	160
Z1.5CR15	190	197	171	175
Z1.5CR20	203	211	182	187
Z3CR10	170	176	153	157
Z3CR15	194	201	174	178
Z3CR20	207	215	185	190

**Table 10:** MSCR test results for CR-modified WMA binders at the maximum temperature achieved (high PG).

Asphalt Binder	PG Temperature (°C)	R_3.2_ (%)	J_nr3.2_ (kPa^−1^)	Traffic Level at 64 °C
S2	64	1.8	2.4	S
S2CR10	70	3.9	2.1	V
S2CR15	76	2.7	3.5	V
S2CR20	82	4.0	3.4	E
S4	70	1.2	4.3	H
S4CR10	76	4.1	2.8	V
S4CR15	76	1.9	4.5	V
S4CR20	76	4.8	2.4	E
Z1.5	64	0.4	3.9	S
Z1.5CR10	70	3.3	2.6	H
Z1.5CR15	70	4.0	2.5	H
Z1.5CR20	76	5.2	2.5	E
Z3	64	0.4	3.9	S
Z3CR10	70	1.5	4.1	H
Z3CR15	70	3.9	2.4	H
Z3CR20	76	4.7	2.7	V

**Table 11:** BBR results for the modified binders.

Asphalt Binder	Low PG Temperature (°C)	m-Value	Creep Stiffness, S (MPa)
S2	−16	0.327	126
S2CR10	−16	0.311	112
S2CR15	−16	0.306	72
S2CR20	−16	0.335	72
S4	−10	0.329	81
S4CR10	−16	0.326	116
S4CR15	−16	0.334	79
S4CR20	−16	0.345	80
Z1.5	−16	0.434	100
Z1.5CR10	−16	0.396	87
Z1.5CR15	−22	0.320	141
Z1.5CR20	−22	0.310	108
Z3	−22	0.310	207
Z3CR10	−16	0.347	180
Z3CR15	−28	0.354	184
Z3CR20	−34	0.318	233

## Data Availability

The data that support the findings of this study are available upon reasonable request.
